# Significance loss brings to extreme self-care related behaviors: the role of interpersonal influence and obsessive (vs. harmonious) passion

**DOI:** 10.3389/fpsyg.2024.1374747

**Published:** 2024-05-09

**Authors:** Federico Contu, Antonio Pierro

**Affiliations:** ^1^“La Sapienza” University of Rome, Rome, Italy; ^2^UniSR-Social.Lab, Vita-Salute San Raffaele University, Milan, Italy

**Keywords:** significance loss, motivational imbalance, interpersonal influence, obsessive vs. harmonious passion, extreme self-care related behaviors

## Abstract

Building on Significance Quest Theory we hypothesized that significance loss feelings can bring people to extreme self-care related behaviors via (a) the susceptibility to interpersonal influence, and (b) the development of a predominance of obsessive (vs. harmonious) passion toward the self-care. To test these hypotheses, we ran one cross-sectional study among voluntary participants (*N* = 401). Results confirmed our hypotheses, suggesting that physical appearance is perceived as a fruitful and useful route to maintain or even restoring ones’ personal sense of significance. Notably, these results shed light on another scope that can be exploited to achieve social significance (i.e., physical appearance) through extremism, and could represent a starting point to design practical intervention to reduce the examined extreme behaviors.

## Introduction

Excessive preoccupation with one’s physical appearance can lead to a myriad of detrimental consequences, both psychologically and socially. Individuals who engage in obsessive appearance management often experience heightened levels of stress, and anxiety (O'Guinn and Faber, 1989). The relentless pursuit of an idealized image can result in a distorted self-perception, fostering feelings of inadequacy and dissatisfaction (Mack, 2018). Moreover, the constant comparison to societal beauty standards can contribute to the development of body dysmorphic disorders, wherein individuals perceive flaws that may not objectively exist, further exacerbating their mental distress. In support of the above-presented notions, the American Society of Plastic Surgery (2018) reported that Americans in 2018 spent more than $16.5 billion on cosmetic plastic surgery.

Socially, the repercussions of obsessive appearance management are profound. Relationships may be strained as individuals prioritize their physical image over genuine connections, and the pursuit of perfection may isolate them from meaningful social interactions (Mack, 2018). This phenomenon also intersects with broader societal issues, perpetuating unrealistic beauty standards that can contribute to discrimination and exclusion based on appearance (Berry, 2007). To address these issues, it is imperative to delve into the underlying factors driving self-care related extreme behaviors. Indeed, by understanding the causes and consequences of obsessive appearance management, we can develop interventions and strategies to promote a more inclusive and compassionate societal ethos, fostering well-being for individuals and communities alike.

### Previous scientific approaches to (extreme) impression management behaviors

The importance of esthetic beauty and physical appearance has been deeply explored by social psychologists (e.g., Swami et al., 2009). Among the previous scientific approaches to extreme impression management behaviors, one of the prominent was drawn on Terror Management Theory (TMT; see for a recent review Greenberg et al., 2014). In a few words, the core assumption of TMT is that individuals strive for preserving the proper self-esteem. That is, when self-esteem is threatened, people are expected in engage in behaviors that should defend (or even restore) the integrity of their self-esteem (see also Steele, 1988). Importantly to our aim, this paradigm was applied to appearance monitoring. More specifically, Goldenberg et al. (2000, Study 3) showed that when self-esteem was threatened and individuals shared the cultural importance of physical appearance, then they engaged in extreme appearance monitoring to meet cultural standards for the body and its appearance.

Nowadays, one of the shapes that extreme physical’s impression management has taken is the one of cosmetic surgery. That is, the “maintenance, restoration or enhancement of one’s physical appearance through surgical and medical techniques” (Swami et al., 2009). In this context, maintaining and boosting appropriate self-esteem is recognized as a crucial factor leading to the pursuit of major medical interventions to control physical appearance (Furnham and Levitas, 2012). However, these presented approaches to impression management were specifically referred to appearance monitoring or cosmetic plastic surgery. Hence, they did not consider ‘extreme’ self-care related behaviors as a unique body of tendencies, joined by the fact of sharing certain features that make them ‘extreme’. Hence, the aim of the present research was to find common motivational factors underlying extreme self-care related behaviors (i.e., ensemble of behaviors akin to each other).

### Motivational imbalance and “extremisms”

The present research, grounded in a socio-motivational framework, aims to examine the hypothesis that extreme self-care related behaviors, encompassing various forms of extremity, arise from a state of motivational imbalance (Kruglanski et al., 2021). Indeed, Kruglanski et al. (2021) posit that when a psychological need takes precedence over all others, resulting in a condition of motivational imbalance, individuals may engage in extreme behaviors to fulfill that particular need. To be more precise, in a recently proposed model of extremism, Kruglanski et al. (2021) assumed that the individuals’ motivational system can be under a condition of “moderation”, or under the one called “imbalance”. When “moderation” occurs, different goals can be pursued and different needs are considered, meaning that peoples’ energies are distributed across various scopes. Moreover, moderation also entails the presence of constraints that prevent people to reach goals at the cost of harming oneself or others.

By contrast, motivational “imbalance” corresponds to a condition in which one need overwhelms the others in importance, thereby removing constraints and becoming the only one that, over time, drives ones’ behaviors to its satisfaction. According to the model, the reasons why motivational imbalance is experienced can be various (e.g., a goal is frustrated, or a need is activated as a response to impending threats). But the key point is that when motivational imbalance is ongoing, people take actions that (a) satisfy the need that caused the imbalance while neglect the others, and (b) do not consider those constraints that should impede to damage oneself or others. As a consequence, most people commonly avoid enacting this kind of behaviors, and they are thus appointed as “extreme” (i.e., not commonly enacted by the most part of the population). This definition of “extreme” can be thus well applied to behaviors of all sorts, as all sorts of desired goals can cause a condition of motivational imbalance. For example, if one is desiring to have a perfect physical prowess, they could engage in healthy diets and physical exercise. However, if that desire will take precedence over all others, extreme eating behaviors and the extreme practicing of sports will be enacted, thus resulting in damages for the self (e.g., family is ignored, or social occasions as lunches or dinners are avoided). As such, also impression management and self-care can be pursued “moderately”, or as a response to a condition of motivational imbalance, thereby resulting in extreme self-care related behaviors. In our research, we have identified the pursuit of significance (Kruglanski et al., 2022) as a fundamental human need that, when dominant over alternative needs, elucidates self-care related extreme behaviors.

### Significance quest theory

Significance Quest Theory (SQT; Kruglanski et al., 2022) holds that all humans strive for significance. That is, a basic and universal need of feeling worthy, valuable, important, and that one matters in face of the other society’s members. It follows that, to be satisfied, the need for significance depends on the “sharing of reality” with others (Higgins, 2019). Specifically, the values and the norms that one must observe are those that are culturally (and socially) dictated. Further, the fact that a person is observing those values and norms must be confirmed and validated by the rest of one’s network or reference group (Webber and Kruglanski, 2016; Kruglanski et al., 2022). More specifically, the network has a dual role. That is, it firstly validates the existence of a significance-lending narrative. Secondly, it overtly rewards with consideration and prestige those who endorse and concretely respect the values embedded in the cultural narrative (e.g., Anderson et al., 2015). Took for example the style in which one wears. For the wearing style to confer social significance, it must be perceived as significance-conferring and aligned with socially established norms (e.g., matching belt and shoe colors). Additionally, one’s network or reference group (e.g., close friends) must validate that these rules bestow significance and that the individual adheres to them, such as by complimenting their taste in style choices.

According to the SQT, the magnitude of one’s quest for significance chronically differs among individuals. However, it also can be situationally activated, thereby depicting differences within individuals depending on the experiences one had. Relevant to this research, one way in which the quest for significance can be activated is through personal or social experiences of *significance loss* (Kruglanski et al., 2014). Experiences of significance loss correspond to feeling lowly valuable and important in the face of others and, as such, they make individuals strive for re-establishing acceptable levels of significance. In line with past research, significance loss is induced through situations that damage one’s sense of mattering (Elliott et al., 2004) and ego-security (Downey et al., 1994; Feldman and Downey, 1994) such as humiliation (Brown and Dutton, 1995; Otten and Jonas, 2014), social rejection (i.e., exclusion; Baumeister et al., 1993), or failure (Brown and Dutton, 1995).

### Significance loss, the susceptibility to interpersonal influence, and the dualistic model of passion

The susceptibility to interpersonal influence (Bearden et al., 1989) is defined as the process through which people’s attitudes are modified because of the influence exerted by others (Cialdini and Sagarin, 2005). More specifically, people can modify their attitudes in function of others because they need to be accepted (i.e., normative influence), or because they do not know what is appropriate in a given situation (i.e., informational influence). According to the three N model of extremism (Kruglanski et al., 2019), one of the key factors to commute the need for significance in extreme actions is the support (or pressure) that one’s network can exert. Indeed, and most importantly, actions can be significance bestowing only if validated by one’s network. It follows that, individuals who are experiencing significance loss will develop susceptibility to interpersonal influence. That is, for example, if in a group of five friends, two of them (named Frank and Hannah) are experiencing significance loss feelings, they will see others’ (positive) judgment as a main route to significance restoration. Hence, they will be susceptible to other friends’ influence because through modifying their behaviors in function of others, Frank and Hannah will likely be approved, and their sense of significance will be restored.

Besides the susceptibility to interpersonal influence, the present research aimed to investigate the role of another potential mediator into the examined process. The dualistic model of passion, proposed by Vallerand et al. (2003), suggests that passion for a particular activity corresponds to a strong engagement in that activity. This model delineates two distinct forms of passion. Harmonious passion involves a robust motivational drive toward an activity while maintaining balance and harmony with other life’s areas. Conversely, obsessive passion entails an intense pursuit of an activity to the extent that other life domains are neglected or even suppressed. For instance, consider a profound passion for the makeup industry. In one scenario, this passion could evolve into a harmonious passion, driving the individual to master various makeup techniques while still allocating time for family. Alternatively, it could manifest as obsessive passion, leading to an exclusive focus on makeup and self-appearance at the expense of other essential life domains.

In this respect, as mentioned above, motivational imbalance depicts a condition in which a need takes precedence over all others (Kruglanski et al., 2021). This meaning that the need that caused the imbalance will direct individuals’ behavior exclusively toward its satisfaction. In this case, if, as we are assuming, the need causing the imbalance is the need for significance (Kruglanski et al., 2022), then people’s behavior will be directed toward activity that are sensed as able to satiate that need, while suppressing others. Hence, individuals will experience a strong motivational push toward the activity experienced as significance-bestowing, thereby neglecting others, and thus developing an obsession for that given activity (Vallerand et al., 2003). In the given example, if an individual experiences motivational imbalance due to a loss of significance and perceives that having an attractive physical appearance is culturally linked to gaining significance, they may develop an obsessive passion for impression management. Aligned with this notion, a substantial body of literature has established a correlation between obsessive passion and ego-insecurity (Donahue et al., 2009; Lafrenière et al., 2011; Rip et al., 2012; Bélanger et al., 2013a), often described as feelings of significance loss (Downey et al., 1994; Feldman and Downey, 1994; Kruglanski et al., 2022). Moreover, research has identified a clear connection between constructs akin to significance loss, and obsessive passion. Lafrenière et al. (2011) discovered that individuals with low self-esteem exhibited heightened levels of obsessive passion. Additionally, Resta et al. (2022) established a positive relationship between ambition, defined as an expression of the quest for significance (DeYoung et al., 2007; Judge and Kammeyer-Mueller, 2012; Jones et al., 2017), and obsessive passion. Notably, recent research conducted by Contu et al. (2023a,b), comprehending two cross-sectional and one longitudinal study, provided evidence supporting the idea that feelings of significance loss could lead individuals to become obsessed with an activity perceived as a means for restoring significance.

Notably, although one could intuitively consider obsessive and harmonious passion as negatively associated, the dualistic model of passion (Vallerand et al., 2003) outlines that they are independent (Schellenberg et al., 2019). That is, similar levels of both HP and OP can be experienced toward the same activity. However, there is also the case in which one of the two passions become predominant over the other. In this latter case, only the predominant passion is expressed, and people are more likely to act and experience outcomes related to the predominant passion (Bélanger et al., 2013b; Schellenberg et al., 2019). Following this approach, in the present research, we focused on the predominant type of passion. Specifically, we focused on the predominance of obsessive over the harmonious passion. Interestingly, this kind of predominance was found to be associated with more negative outcomes (Schellenberg et al., 2019) and with radical and deviant behaviors (Bélanger et al., 2013b; Chirico et al., 2021).

### Significance loss, obsessive passion, and the willingness to the extreme

A lot of past research identified the quest for significance as a main fundamental precursor of the willingness to extremism (Da Silva et al., 2023). Specifically, these studies supported the basic principle that when the need for significance is activated – and thus motivational imbalance is experienced – extremism is used to fulfill this need (see also Kruglanski et al., 2014, 2017, 2018). This prediction has been tested through several research in various scopes. For example, Adam-Troian et al. (2020) found a positive association between quest for significance and extreme political activism among Yellow Vest in France. Further, Jasko et al. (2017) expanded these results also to political and religious radicals and we are plenty of others successful applications of the SQT to the extreme behaviors arena (e.g., Kruglanski et al., 2009, 2019; Dugas et al., 2016). Notably, the quest for significance predicted extremism also within individuals’ private life-scopes. For instance, Chirico et al. (2021) found that participants (non-professional athletes) striving to feel significant were prone to doping consumption to improve their physical performances and, in so doing, satiating their need for significance.

Importantly, the link between significance and extremism was not found always to be direct (Da Silva et al., 2023). Indeed, a series of studies identified an important mediator of this process – i.e., the obsessive passion. As mentioned above, obsessive passion can be induced by psychological states close to that of loss of significance (Resta et al., 2022; Contu et al., 2023a). Moreover, Resta and colleagues found that although ambition was predictive of both obsessive and harmonious passion, ambitious people showed an enhanced willingness to engage in extreme behaviors only when they developed an obsessive (but not harmonious) passion toward a specific goal. Relevantly, Contu et al. (2023a,b) replicated these results with regard to the romantic relationships arena. That is, they found that general feelings of significance loss were associated to obsessive and harmonious passion toward romantic partners and relationships but only the obsessive passion brought participants to the proneness to (a) self-sacrifice for the relationship, and (b) intrusive actions toward the romantic partner.

### The present research

As presented above, significance loss feelings have been linked to a myriad of those extreme behaviors that were sensed as significance-bestowing (Resta et al., 2022; Contu et al., 2023a; see for a review Da Silva et al., 2023). Moreover, obsessive passion has been shown to be an important mediator in the process that bring people from insignificance to “romantic” extremism (Contu et al., 2023a), or to extremism enacted with respect to an activity that was important within individuals’ lives (Resta et al., 2022). Further, supporting the three N model of extremism (Kruglanski et al., 2009), past research showed that social pressure to conformity augment the likelihood of people feeling insignificant to act extremely within the political arena (Jasko et al., 2020). However, these research investigating the role of the quest for significance in predicting various kinds of extremism covered scopes as political and religious ideologies (e.g., Jasko et al., 2017; Adam-Troian et al., 2020), or romantic relationships (Contu et al., 2023a); without paying attention to extreme behaviors related to the self.

Aiming to address this gap in knowledge, we applied Significance Quest Theory (Kruglanski et al., 2022) to the self-care arena, hypothesizing that when people feel a *general* lack of self-worth, they are more likely to be susceptible to others’ influence and, as a consequence, to develop a predominance of obsessive (vs. harmonious) passion toward their self-care, which, in turn, would enhance peoples’ willingness to extreme self-care related behaviors. Hence, more specifically, we proposed a sequential mediation hypothesis where (a) significance loss was the main predictor, (b) the susceptibility to interpersonal influence was the first mediator, (c) the predominance of obsessive (vs. harmonious) passion was the second mediator, and (d) the tendency to act extreme self-care related behaviors was the dependent variable. Moreover, given the nature of our hypothesis, we also expected that significance loss would have had an indirect effect on the tendency to act extreme self-care related behaviors via the two mediators.

## Methods

### Participants, design, and procedure

To determine the minimum sample size to detect the indirect effects of a serial mediation model (two mediators), we used the online tool “Monte Carlo Power Analysis for Indirect Effects” by Schoemann et al. (2017). Assuming medium effect sizes (*r* = 0.30), the confidence level set at 95 percent, and power set at 0.80, 5,000 Monte Carlo simulations indicated a minimum sample size of 261 participants to detect the indirect effect of loss of significance on extreme self-care related behaviors via the susceptibility to others’ influence and the predominance of obsessive (vs. harmonious) passion.

To test our hypothesis, we enrolled 401 Italian adults (22.1% males; *M*_age_ = 28.36, *SD*_age_ = 9.26) in a correlational study. Participants were recruited online and volunteered in the study through an online survey hosted by Google Forms. More specifically, the link through which participants could reach the survey was spread through common social network (i.e., Whatsapp, Telegram, Facebook, Instagram). After giving their informed consent, each participant filled out an online questionnaire aimed at assessing the measures of interest (as described below). All items were administered in Italian (i.e., items are a linguistics adaptation from English to Italian), but in the following section we provide examples of the employed items translated into English.

## Measures

### Significance loss

Significance loss was assessed with a five-item measure based on Significance Quest Theory (Kruglanski et al., 2022) and already used in recent research by Contu et al. (2023a,b). Participants had to respond on a seven-point Likert scale ranging from 1 (do not agree at all) to 7 (very strongly agree). Example items: “I feel humiliated”; “I feel disrespected.” (α = 0.87).

### Susceptibility to normative and influence interpersonal influence

Participants’ susceptibility to interpersonal influence was measured through an eight-item scale adapted to apply to self-care from the Consumer Susceptibility to Interpersonal Influence Scale (Bearden et al., 1989). Participants rated each item on a seven-point scale ranging from 1 (Not agree at all) to 7 (Very strongly agree). Examples items: “It is important to me that others like my external appearance”; “I often ask my friends which are the best self-care products.” (α = 0.91).

### Obsessive and harmonious self-care related passion

Participants were asked to respond to a total of 12 items (6 for the obsessive and 6 for the harmonious passion) on a seven-point Likert scale ranging from 1 (do not agree at all) to 7 (very strongly agree). Items were an Italian adaptation to the self-care arena of the original Passion Scale (Vallerand et al., 2003). Sample items are: “I am emotionally dependent on whether or not I can take care of my appearance.”; “Taking care of my appearance reflects the qualities I like about myself.” Reliability was α = 0.91 for the obsessive passion, and α = 0.75 for harmonious one. We then aggregated items to compose measures of harmonious and obsessive passion. Eventually, we subtracted scores of harmonious passion from scores of the obsessive one. In so doing, we obtained a measure for which high scores indicated a predominance of obsessive (vs. harmonious) passion (for similar approaches see Bélanger et al., 2013b; Schellenberg et al., 2019).

### Extreme self-care related behaviors

Self-care related extreme behaviors were measured with a seven-item scale adapted to self-care arena from the Compulsive Buying Scale (Valence et al., 1988). Participants rated each item on a seven-point scale ranging from 1 (Not agree at all) to 7 (Very strongly agree). Example items: “I spend more money than I should on my appearance care”; “The idea of leaving the house without having taken care of my appearance disturbs me a lot.” (α = 0.89).

### Data analysis

Analyses were conducted using SPSS Statistic version 27.0. To test the hypothesized sequential mediation model, we used PROCESS v4.0, Model 6 (Hayes, 2018) and 95% bias-corrected confidence intervals were obtained with 5,000 bootstrap samples. More specifically, we tested a model in which general significance loss feelings were the main predictor, susceptibility to interpersonal influence was the first mediator, the predominance of self-care related obsessive (vs. harmonious) passion was the second one, and the tendency to extreme self-care related behaviors the dependent variable. Eventually, participants’ age and gender were considered as covariates, and they were thus inserted as regression’s terms in the sequential mediation model (Table 1 shows covariates’ regression parameters). Pearson’s bivariate correlations and descriptive statistics are summarized in Table 2. Of particular interest for our aim, significance loss resulted positively and significantly correlated with the susceptibility to interpersonal influence *r*(400) = 0.175, *p* < 0.001, which, in turn, positively and significantly correlated with the predominance of obsessive (vs. harmonious) passion *r*(400) = 0.147, *p* = 0.003. Eventually, the predominance of obsessive (vs. harmonious) passion positively and significantly correlated with extreme self-care related behaviors *r*(400) = 0.307, *p* < 0.001. Also, we performed an Hotelling’s t test that revealed, as hypothesized, that the correlations between significance loss and obsessive passion [*r*(400) = 0.226, *p* < 0.001] was stronger than that between significance loss and harmonious passion [*r*(400) = 0.092, *p* = 0.065], *t*(398) = 3.32, *Z* = 3.28.

**Table 1:** Covariates’ regression table.

Dep	Pred	Estimate	SE	CI95%-L	CI95%-U	*z*	*p*
SUS	Gender	0.277	0.149	−0.0165	0.5710	1.84	0.064
SUS	Age	−0.026	0.006	−0.0396	−0.0133	−3.95	< 0.001
ΔP	Gender	0.071	0.131	−0.1865	0.3299	0.54	0.586
ΔP	Age	−4.58e-4	0.005	−0.0122	0.0112	−0.07	0.939
SCB	Gender	0.070	0.121	−0.1670	0.3079	0.58	0.561
SCB	Age	−0.001	0.005	−0.0125	0.0089	−0.32	0.746

SUS, Susceptibility to interpersonal influence; ΔP, Predominance of Obsessive vs. Harmonious passion; SCB, Extreme self-care related behaviors. Gender coded as 0, male; 1, female.

**Table 2:** Descriptives statistics and bivariate correlations.

	LOSS	SUS	ΔP	OP	HP	SCB	Age	M (*SD*)
LOSS	(0.87)							2.70(1.34)
SUS	0.175***	(0.91)						2.93 (1.29)
ΔP	0.192***	0.147**	—					−0.76 (1.11)
OP	0.226***	0.493***	0.581***	(0.91)				2.95 (1.43)
HP	0.092	0.453***	−0.233***	0.657***	(0.75)			3.71 (1.20)
SCB	0.209***	0.637***	0.307***	0.696***	0.548***	(0.89)		2.41 (1.35)
Age	−0.034	−0.192***	−0.030	−0.137**	−0.135**	−0.133**	—	28.26 (9.26)
Gender	0.123	0.102	0.059	0.209	0.195	0.101	0.039	—

Reliability is displayed in parenthesis. LOSS, Loss of significance; OP, Obsessive passion; HP, Harmonious passion; ΔP, Predominance of Obsessive vs. Harmonious passion; SUS, Susceptibility to interpersonal influence; SCB, Extreme self-care related behaviors. Gender coded as 0, male; 1, female.

## Results

**Figure 1:**
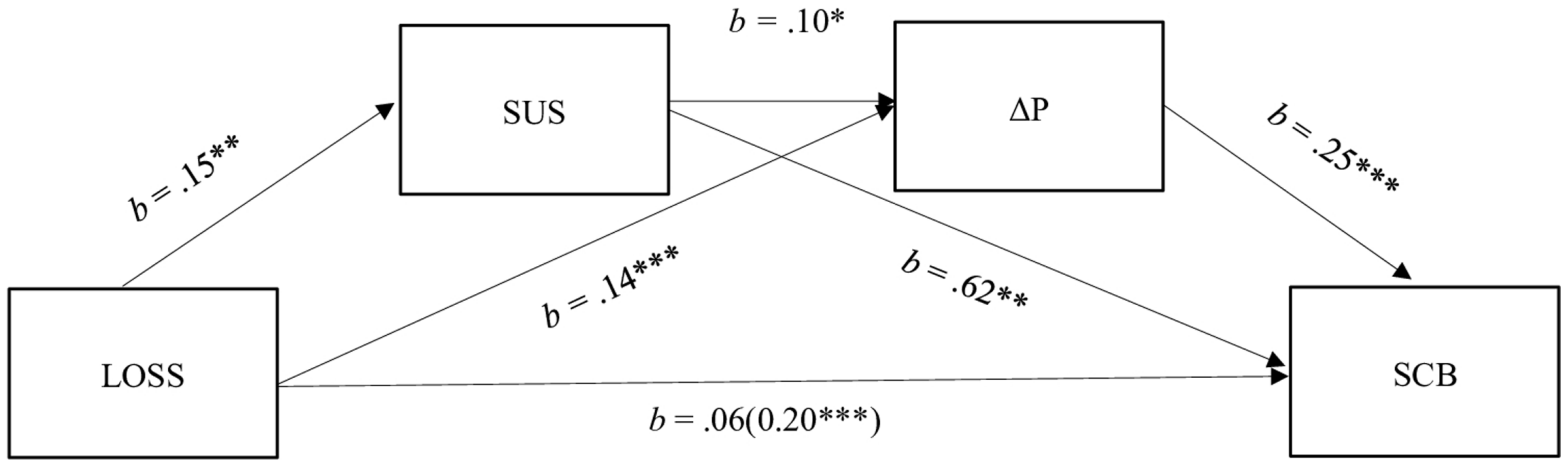
A model showing the effects of significance loss on extreme self-care related behaviors via the susceptibility to interpersonal influence and the predominance of obsessive (vs. harmonious) passion.

As shown in Figure 1, the results of the sequential mediation revealed that significance loss positively predicted the susceptibility to interpersonal social influence [*b* = 0.15, SE = 0.05, *t* = 3.24, *p* = 0.001, (95%CI = 0.06, 0.24)], which, in turn, positively predicted the predominance of obsessive (over harmonious) passion [*b* = 0.10, SE = 0.04, *t* = 2.24, *p* = 0.025, (95%CI = 0.01, 0.18)]. Eventually, the predominance of obsessive (vs. harmonious) passion was positively associated with participants’ tendency to extreme self-care related behaviors [*b* = 0.17, SE = 0.06, *t* = 3.06, *p* = 0.002, (95%CI = 0.06, 0.28)]. Most importantly, all the estimated indirect effects were positive and significant. Indeed, significance loss positively predicted the tendency to extreme self-care related behaviors via (a) the susceptibility to interpersonal influence [Effect = 0.09, bootSE = 0.04, (95%CI = 0.02, 0.16)], (b) the predominance of obsessive (vs. harmonious) passion [Effect = 0.04, bootSE = 0.01, (95%CI = 0.01, 0.06)], and (c) both the two mediators [Effect = 0.004, bootSE = 0.002, (95%CI = 0.001, 0.009)]. Hence, also the total indirect effect was positive and significant [Effect = 0.13, bootSE = 0.04, (95%CI = 0.05, 0.21)]. Notably, the total effect of the significance loss on extreme self-care related behaviors was positive and significant [*b* = 0.20, SE = 0.05, *t* = 3.97, *p* < 0.001, (95%CI = 0.10, 0.29)]. By contrast, the direct effect of significance loss on extreme self-care related behaviors was not significant (*p* = 0.104), thereby suggesting that the effect of significance loss on extreme self-care related behaviors was fully mediated by the mediators. Eventually, results revealed that significance loss was positively associated with the predominance of obsessive (vs. harmonious) passion [*b* = 0.14, SE = 0.04, *t* = 3.36, *p* < 0.001, (95%CI = 0.06, 0.22)]. Such as the susceptibility to interpersonal influence with respect to extreme self-care related behaviors [*b* = 0.62, SE = 0.04, *t* = 15.25, *p* < 0.001, (95%CI = 0.54, 0.70)].

Given the correlational nature of our data, we estimated an alternative model in which the tendency to extreme self-care related behaviors was the main predictor. Significance loss was the dependent variable, the predominance of obsessive (vs. harmonious) passion was the first mediator, and the susceptibility to interpersonal influence was the second mediator. As expected, analyses revealed that neither the indirect effect of extreme self-care related behaviors on significance loss via the two above-mentioned mediators [Effect = −0.001, bootSE = 0.002, (95%CI = −0.006, 0.002)], nor the total indirect effect [Effect = 0.09, bootSE = 0.05, (95%CI = −0.001, 0.18)] were significant. Hence, we decided to accept the first model (the one we hypothesized) we estimated.

## Discussion

Extreme self-care related behaviors are directly associated with a series of severe psychological and physical consequences (e.g., O'Guinn and Faber, 1989; Berry, 2007; Mack, 2018). For this reason, the main aim of the present research was to identify the motivational basis of this kind of behaviors. To achieve this important goal, we applied significance quest theory (Kruglanski et al., 2022) to the realm of extreme self-care related behaviors. The hypothesis we derived from that theory was that significance loss feelings would be associated with the tendency to act extreme self-care related behaviors sequentially via (a) the susceptibility to interpersonal influence, and (b) the predominance of the obsessive (vs. the harmonious) passion. Importantly, the analyses that we ran confirmed our expectations.

These results are basically in accordance with a series of past studies on significance quest theory and its application to extremism (see for a review Da Silva et al., 2023). Importantly, the role of the predominance of obsessive (vs. harmonious) passion in the process that from significance loss feelings motivates self-care related extremism has been confirmed. In this respect, these results directly corroborate previous and recent studies on extremism (e.g., Chirico et al., 2021). Further, the present findings are also aligned with previous research that showed – longitudinally and cross-sectionally – that the obsessive (and not the harmonious) passion is associated with extremism with respect to general activities (Resta et al., 2022), and romantic relationships (Contu et al., 2023a). Relevantly, also the results entailing the mediational role of the susceptibility to interpersonal influence represent a confirmation – and an expansion – of past research. Indeed, the ‘network’ pressure to conformity was already showed to magnify the effect of significance loss feelings on extremism (Jasko et al., 2020), but the present research is the first to have considered individuals’ susceptibility – and not received pressure – to interpersonal influence.

### Theoretical implications

The present research represents the first application of the significance quest theory (Kruglanski et al., 2022) to the realm of extreme self-care related behaviors. That is, the present findings enrich the socio-motivational literature on extremism and expand the explicative power of significance quest theory to the examined field. Results from this study thus suggest and confirm that taking care of physical appearance is a fruitful strategy to restoring ones’ general sense of personal significance. To summarize, looking back at the past literature, we now know that significance quest theory can be successfully applied to explain extremism into various – and diversified – fields, including political activism (Jasko et al., 2020), religious ideologies (Adam-Troian et al., 2020), sports (Chirico et al., 2021), romantic relationships (Contu et al., 2023a), self-care, and workplaces (Contu et al., 2023b).

In this respect, two main considerations can be done. First, significance quest theory (Kruglanski et al., 2022) appears useful to explain and predict people’s willingness to act extremely both (a) when the extreme behavior is directed toward (and thus can damage) oneself (e.g., extremism within the workplace or self-care related extremism), and (b) when the extreme behavior is directed toward (and thus can damage) another person (e.g., extremism acted toward a romantic partner).

Second, the fact that the significance quest theory can be successfully applied to extremism within various and variegate fields supports the idea that significance restoration can be achieved by acting extremely within a life-scope that is different from the one in which the significance loss was generated. This idea is not new, and it was already presented within the theory itself (Kruglanski et al., 2022). Research carried out drawing from other theoretical perspectives (e.g., terror management theory, Greenberg et al., 2014; symbolic self-completion, Wicklund and Gollwitzer, 1981) indirectly corroborated this hypothesis (e.g., Sciara et al., 2023; Contu et al., 2023c). However, it exists only one study that directly tested this hypothesis within the theoretical framework of the significance quest theory. And, specifically to this case, Contu et al. (2023b, study 1) found that feelings of significance loss originated within one’s romantic relationship can bring people to act extremely within the workplace to restore their significance. Interestingly, it was found that also the opposite was true. Indeed, feelings of significance loss originated within the workplace were associated to the willingness to act extremely toward the proper romantic partner to restore the sense of significance.

### Limits

Given the correlational nature of our study, we were not able to provide evidence about the casual relationship among the constructs we studied in this research. In this respect, we have only longitudinal evidence about the fact that significance loss can predict obsessive (romantic) passion and that obsessive (romantic) passion can predict, in turn, extremism within romantic relationships (Contu et al., 2023a). However, these results were obtained with respect to the romantic relationships arena and were not experimental. Indeed, only few studies experimentally demonstrated that significance loss motivates people to extremism (e.g., Dugas et al., 2016). But, however, these were not carried out within the self-care context and thus are not conclusive for our aim. Hence, future research should address this limit by implementing true experiments, thereby providing casual evidence about the relationship among significance loss, obsessive passion, susceptibility to interpersonal influence, and extreme self-care related behaviors.

## Data availability statement

The raw data supporting the conclusions of this article will be made available by the authors, without undue reservation.

## Ethics statement

The studies involving humans were reviewed and approved by the Ethics Committee of the Department of Social and Developmental Psychology at “La Sapienza” University of Rome (protocol N. 572). The studies were conducted in accordance with the local legislation and institutional requirements. The participants provided their written informed consent to participate in this study.

## Author contributions

FC: Data curation, Formal analysis, Funding acquisition, Methodology, Writing – original draft. AP: Conceptualization, Data curation, Formal analysis, Funding acquisition, Methodology, Supervision, Writing – review & editing.
